# Rapid development of an updated mRNA vaccine against the SARS-CoV-2 Omicron variant

**DOI:** 10.1038/s41422-022-00626-w

**Published:** 2022-02-14

**Authors:** Na-Na Zhang, Rong-Rong Zhang, Yi-Fei Zhang, Kai Ji, Xiao-Chuan Xiong, Qian-Shan Qin, Peng Gao, Xi-Shan Lu, Hang-Yu Zhou, Hai-Feng Song, Bo Ying, Cheng-Feng Qin

**Affiliations:** 1 Department of Virology, State Key Laboratory of Pathogen and Biosecurity, Beijing Institute of Microbiology and Epidemiology, Academy of Military Medical Sciences, Beijing, China; 2 School of Medicine, Tsinghua University, Beijing, China; 3 Kunming University of Science and Technology, Kunming, Yunnan China; 4 Suzhou Abogen Biosciences Co., Ltd., Suzhou, Jiangsu China; 5 School of Life Sciences, Fujian Agriculture and Forestry University, Fuzhou, Fujian China; 6 Institute of Systems Medicine, Chinese Academy of Medical Sciences & Peking Union Medical College, Beijing, China; 7 Research Unit of Discovery and Tracing of Natural Focus Diseases, Chinese Academy of Medical Sciences, Beijing, China

**Keywords:** Biological techniques, Immunology

Dear Editor,

Since the declaration of public Health Emergency of International Concern (PHEIC) by the WHO, the COVID-19 pandemic, caused by the severe acute respiratory syndrome coronavirus 2 (SARS-CoV-2), has led to over 300 million confirmed cases with more than 5 million deaths in the past 2 years. On top of that, SARS-CoV-2 continues evolving into many variants, and many of these variants with evidence to enhance viral transmissibility, adaptiveness, infectivity, and/or to escape from host immune response are classified as variants of concerns (VOC).^1^ Since the outbreak of the pandemic, five VOCs, including Alpha, Beta, Gamma, Delta, and Omicron have been verified by the WHO.

The newest SARS-CoV-2 VOC, Omicron (also known as B.1.1.529) designated by WHO was first reported in South Africa in November 2021. In a few weeks, Omicron has thrived throughout the world and became the predominantly circulating strain in most continents. Remarkably, Omicron carries an unprecedented number of mutations/deletions/insertion (over 30) in the spike (S) protein as well as the receptor binding domain (RBD), the main target of the host immune responses and vaccine development. Many of these mutations, e.g., K417Y, E484A, N501Y, D614G, P681H, have been identified in other VOCs and are predicted to affect neutralization epitopes.^2,3^ Indeed, accumulated evidence has demonstrated that the Omicron variant can largely escape from vaccination, convalescent sera and most approved monoclonal antibodies.^4,5^ For example, the most potent mRNA vaccines, BNT162b2 from Pfizer-BioNTech and mRNA-1273 from Moderna, also showed significant reduction in neutralization antibody titers against the Omicron variant.^6–8^

Previously, we have developed a Lipid nanoparticle (LNP)-encapsulated mRNA vaccine ARCoV,^9^ which is at the final stage of a multi-regional phase 3 clinical trial (NCT04847102). Distinct from BNT162b2 and mRNA-1273, the two full-length S proteins-based mRNA vaccines, ARCoV encodes the RBD of the wild-type (WT) SARS-CoV-2 S protein. As there are 15 variant mutations in Omicron RBD (Fig. 1a), it is interesting to assess the neutralizing activity of serum samples from vaccinees of ARCoV against the Omicron variant. To do so, a panel of serum samples (*n* = 11) from participants in the phase 1 clinical trial^10^ were analyzed for their neutralizing Ab titers using VSV-based pseudovirus. All samples were collected on day 14 post two-dose immunization with 15 µg of ARCoV. As shown in Fig. 1b, most samples (8/11, 72.7%) retained low but detectable neutralization activity against Omicron, with a 47-fold reduction in geometric mean titers (GMTs) against Omicron compared to the WT strain (GMT 1440.87 to 30.67). This observation is consistent with other studies with convalescent or vaccinee sera,^5–8^ suggesting the immune escape capability of Omicron.

**Fig. 1:**
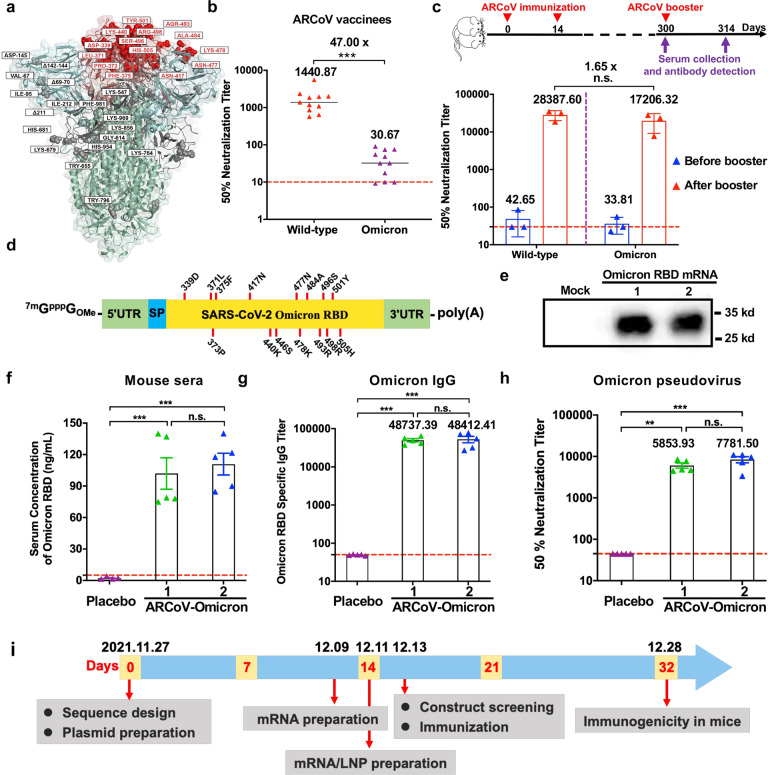
Development and characterization of an updated RBD-based mRNA vaccine against Omicron. **a** Structural model of amino acid mutations in the Spike protein of the Omicron variant of SARS-CoV-2. The model was built using SWISS-MODEL^15^ with a structure of SARS-CoV-2 spike protein (PDB: 6 ZGE^16^) as the template model and visualized with PyMOL(v2.5.0). The RBD, NTD, and S2 region of Spike protein were colored by red, blue and green, respectively. All mutations in the RBD were highlighted in darker color, and mutations or deletions in other region of Spike protein were colored in gray. **b** The 50% neutralization titer of sera from ARCoV vaccinees (*n* = 11) were analyzed using VSV-based pseudovirus for WT and Omicron, respectively. The red dashed lines indicate the detection limit of the assay. Data are shown as means ± SEM and analyzed using unpaired *t*-test (***P < 0.001). **c** Neutralization assay after a booster ARCoV vaccination in mice. Groups of 8–9-month female BALB/c mice were intramuscularly immunized and boosted with 10 μg ARCoV (*n* = 3), and sera were detected at the indicated time points. The red dashed lines indicate the detection limit of the assay. Data are shown as means ± SEM and analyzed using a one-way ANOVA with multiple comparisons tests (n.s., not significant). **d** Schematic representation of the mRNA construct encoding Omicron RBD. The mutation sites were indicated with red lines. **e** Omicron RBD protein expression from mRNA in Huh-7 cells. Cells were transfected with Omicron RBD-encoding mRNAs, and the supernatants was detected by Western blotting assay 24 h after transfection. **f** In vivo expression of ARCoV-Omicron in mice. Groups of 6–8-week female ICR mice (*n* = 5) were intravenously inoculated with ARCoV-Omicron, and PBS (*n* = 4) was used as Placebo. The serum concentration of Omicron RBD was measured by ELISA. Data are shown as means ± SEM and analyzed using one-way ANOVA with multiple comparisons tests (n.s., not significant; ***P < 0.001). **g**, **h** The immunogenicity of ARCoV-Omicron in mice. Groups of 6–8-week female ICR mice (*n* = 5) were immunized intramuscularly with two doses of 10 μg of ARCoV-Omicron at 7-day interval. Sera were collected at 14 days after initial immunization and subjected to IgG and neutralization antibody assays. The red dashed lines indicate the detection limit of the assay. Data are shown as means ± SEM and analyzed using a one-way ANOVA with multiple comparisons tests (**P < 0.01, ***P < 0.001). **i** Timeline for the rapid development of ARCoV-Omicron mRNA vaccine.

A third dose of mRNA vaccination (booster) has been widely used and well evidenced to induce more robust antibody response and improve vaccine efficacy against VOCs.^11–13^ To make sure the potential impact of a homologous booster of ARCoV, groups of 8–9-month female BALB/c mice that have received two doses of ARCoV were further boosted with a third dose ARCoV at day 300 post prime immunization (Fig. 1c). Remarkably, a booster immunization readily induced the production of neutralization antibody, and the GMTs against WT and Omicron increased to 28387.66 and 17206.32, respectively. Interestingly, only 1.65-fold reduction in GMTs against WT and Omicron was observed, and the difference is not statistically significant (Fig. 1c). This result highlights the invaluable benefits of a homologous booster vaccination and supports further validation in clinical trials.

Considering the reduced neutralizing activities of sera from ARCoV vaccinees against Omicron variant, a new mRNA vaccine that directly targets the Omicron RBD will be critical. We therefore took an immediate action to start the effort of developing an Omicron mRNA vaccine based on our established LNP-mRNA vaccine platform. Briefly, the mRNAs encoding the Omicron RBD (Fig. 1d) were prepared and processed into the final LNP formulation as previously described.^9^ The most potent two mRNA constructs, named Omicron/1 and Omicron/2, were selected from a total of 18 mRNAs with different untranslated regions (UTRs) and codon optimizations based on their in vitro expression levels detected by enzyme linked immunosorbent assay (ELISA) (Supplementary information, Fig. S1). Western blotting results demonstrated that recombinant Omicron RBD proteins were expressed and secreted in the supernatants of Huh-7 cells transfected with both mRNAs (Fig. 1e). Immunofluorescence staining results further confirmed that both RBD proteins could be recognized by an Omicron RBD-reactive monoclonal antibody (Supplementary information, Fig. S2). Upon intravenous injection, both LNP-formulated mRNAs, ARCoV-Omicrons, are potent in producing Omicron RBDs in mouse sera (Fig. 1f). After two doses of intramuscularly immunizations at 7-day interval, robust IgG antibodies as well as neutralization antibodies were readily induced by the two ARCoV-Omicrons (Fig. 1g, h) at 14 days post initial immunization. The antibody titers induced by ARCoV-Omicrons were comparable to the original mRNA vaccine ARCoV. It should be mentioned that a 0,7-immunization schedule was utilized in this study to accelerate research and development process, and a more robust antibody response can be expected with a regular 0,14-immunization schedule. The final clinical grade mRNA vaccine is currently being manufactured in a Good Manufacturing Practice (GMP) factory.

Overall, our data presented here clearly demonstrate that a third dose of ARCoV would probably lead to a sharp increasement in neutralization antibodies not only against the WT SARS-CoV-2 but also the newly Omicron variant. Homologous booster vaccination with ARCoV represents a rational strategy in response to the Omicron emergency. More importantly, the continuously evolving SARS-CoV-2 calls for the most flexible and deployable mRNA vaccine platform. Starting from the Omicron RBD sequence, it took 32 days to obtain the first set of immunogenicity results from animal studies (Fig. 1i), and clinical grade vaccine will be ready in less than 3 weeks. As ARCoV-Omicron was produced under the same procedure and release specification as its original version ARCoV,^14^ ARCoV-Omicron can be stored at refrigerator temperature for at least 6 months. To our knowledge, this is the first mRNA vaccine candidate against the Omicron variant that has been validated in animals. We are approaching to clinical trials to test the safety and efficacy of ARCoV-Omicron.

## Supplementary information

Supplementary Methods

Supplementary information, Fig. S1

Supplementary information, Fig. S2

## References

[1] Plante JA (2021). Cell Host Microbe.

[2] He X, Hong W, Pan X, Lu G, Wei X (2021). Med. Commun..

[3] Cao Y (2021). Nature.

[4] Planas D (2021). Nature.

[5] Lu, L. et al.. *Clin. Infect. Dis*. ciab1041 (2021).

[6] Liu L (2021). Nature.

[7] Cele S (2021). Nature.

[8] Cameroni E (2021). Nature.

[9] Zhang NN (2020). Cell.

[10] Chen GL (2022). Lancet Microbe.

[11] Saciuk Y, Kertes J, Shamir Stein N, Ekka Zohar A (2022). J. Infect. Dis..

[12] Ai J (2022). Cell Res..

[13] Corbett, K.S. et al. *bioRxiv*, 10.1101/2021.08.11.456015 (2021).

[14] Zhao H (2021). Signal Transduct. Target Ther..

[15] Waterhouse A (2018). Nucleic Acids Res..

[16] Wrobel AG (2020). Nat. Struct. Mol. Biol..

