# Supplementary material for: Rapid development of an updated mRNA vaccine against the SARS-CoV-2 Omicron variant
Source: Cell Res. 2022 Feb 14;32(4):401–3. doi: 10.1038/s41422-022-00626-w (PMC8853430; doi:10.1038/s41422-022-00626-w)
Supplement: Supplementary file 1 — Supplementary Methods [file 41422_2022_626_MOESM1_ESM.pdf]

**Supplementary information, Materials and Methods**

***Ethic statement***

The phase I clinical trial of ARCoV mRNA vaccine (ChiCTR2000034112) was approved by the IRB of Shulan (Hangzhou) Hospital (YW2020-031-01). All animal experiments were approved by the Experimental Animal Committee of Laboratory Animal Center, AMMS (approval number: IACUC-IME-2021-022).

***Cells***

HEK-293T (ATCC; CRL-11268), Huh-7 (JCRB; 0403), and Vero cells (ATCC; CCL-81) were propagated in complete growth in Dulbecco's minimal essential medium (DMEM; Thermo Fisher Scientific) supplemented with 10% fetal bovine serum (FBS; Thermo Fisher Scientific) and penicillin (100 U/ml)-streptomycin (100 μg/ml) (Thermo Fisher Scientific).

***Sequence alignment and structural modeling***

Structural modeling was built using SWISS-MODEL[1] (https://swissmodel.expasy.org) with 6 ZGE[2] as the template model and visualized with PyMOL (v2.5.0). All mutations on the spike protein were compared with the reference sequence (NC_045512).

***mRNA preparation and characterization***

All optimized DNA sequences encoding Omicron RBD were synthesized (Tsingke Biotechnology) and cloned into the plasmid vectors as described previously.[3]

The mRNAs were produced in vitro using T7 RNA polymerase-mediated transcription from linearized DNA templates.

All Omicron RBD-encoding mRNAs (5 μg) were transfected into HEK-293T, Huh-7 or Vero cells using Lipofectamine$^{TM}$ 2000 (Thermo Fisher Scientific) following the manufacturer's instruction, and the supernatant and lysates were collected and subjected to ELISA, Western blotting, and immunofluorescent staining assays, respectively.

The Omicron RBD concentration was measured by ELISA as previously described.[3] Breifly, 96-well microtiter plates coated with human ACE2 (Sino Biological) were incubated with culture supernatants or mouse sera in serial dilutions. The absorbance at 450/620 nm was measured and accurate quantification were conducted using SpectraMax iD3 (Molecular Devices).

***LNP-mRNA formulation***

Lipid-nanoparticle (LNP) formulations were prepared using the same procedure for ARCoV.[3] Briefly, lipids were dissolved in ethanol containing an ionizable lipid, 1, 2-distearoyl-sn-glycero-3-phosphocholine (DSPC), cholesterol and PEG-lipid (with molar ratios of 48:10:40.5:1.5). The lipid mixture was combined with 20 mM citrate buffer (pH4.0) containing mRNA at a ratio of 1:2 through a T-mixer. Formulations were then diafiltrated against 10x volume of PBS (pH7.4) through a tangential-flow filtration (TFF) membrane with 100 kD molecular weight cut-offs (Sartorius Stedim Biotech). The final formulations were tested for mRNA purity, mRNA encapsulation efficiency, particle size, and size distribution with the same release specification as ARCoV.[3]

***Omicron RBD expression in vivo***

Groups of 6-8 weeks female ICR mice (n=5) were intravenously inoculated with ARCoV-Omicrons (1 mg/kg), and PBS (n=4) was used as Placebo. Sera were collected 6 hours post injection, and Omicron RBD concentration was determined by ELISA as described above.

***Mouse vaccination***

For ARCoV booster immunization, groups of female BALB/c mice aged 8-9 months (n=3) were intramuscularly immunized with 10 μg ARCoV at day 0, 14, and boosted at day 300. The blood samples were collected before and 14 days after the booster dose for detection of neutralizing antibodies against Wild-type and Omicron SARS-CoV-2.

For ARCoV-Omicron immunization, groups of female ICR mice aged 6-8 weeks (n=5) were immunized intramuscularly with 10 μg ARCoV-Omicron at day 0 and 7. Blood was collected at 14 days after initial immunization for Omicron specific IgG and pseudovirus-based neutralizing antibodies as described below.

***Omicron specific IgG and neutralization antibody detection***

The Omicron RBD-specific IgG antibody titers were detected by a modified ELISA as previously described.[3] The VSV-based pseudovirus neutralization assay was performed as previously described.[3] Briefly, heat-inactivated serum samples in serial dilutions were mixed with pseudoviruses carrying the spike of SARS-CoV-2 Wild-type or Omicron variant (Vazym, China) before adding to Huh-7 cells in 96-well white plate. Luciferase activity was measured using GloMax® 96 Microplate Luminometer (Promega). The 50% neutralization titer ($NT_{50}$) value were calculated by fitting a non-linear five-parameter dose-response curve with GraphPad Prism version 9.0.

***Statistical analysis***

Data were presented as the mean ±SEM. The difference between any two groups were determined by unpaired parametric *t*-test or one-way ANOVA with multiple comparisons tests depending on the distribution of the data. All graphs were generated with GraphPad Prism version 9.0 software.

## REFERENCES

1 Waterhouse, A. et al. Nucleic Acids Res 46, W296-W303 (2018).
2 Wrobel, A. G. et al. Nat Struct Mol Biol 27, 763-767 (2020).
3 Zhang, N. N. et al. Cell 182, 1271-1283 e1216 (2020).
