# Supplementary material for: Rapid development of an updated mRNA vaccine against the SARS-CoV-2 Omicron variant
Source: Cell Res. 2022 Feb 14;32(4):401–3. doi: 10.1038/s41422-022-00626-w (PMC8853430; doi:10.1038/s41422-022-00626-w)
Supplement: Supplementary file 2 — Supplementary information, Fig. S1 [file 41422_2022_626_MOESM2_ESM.pdf]

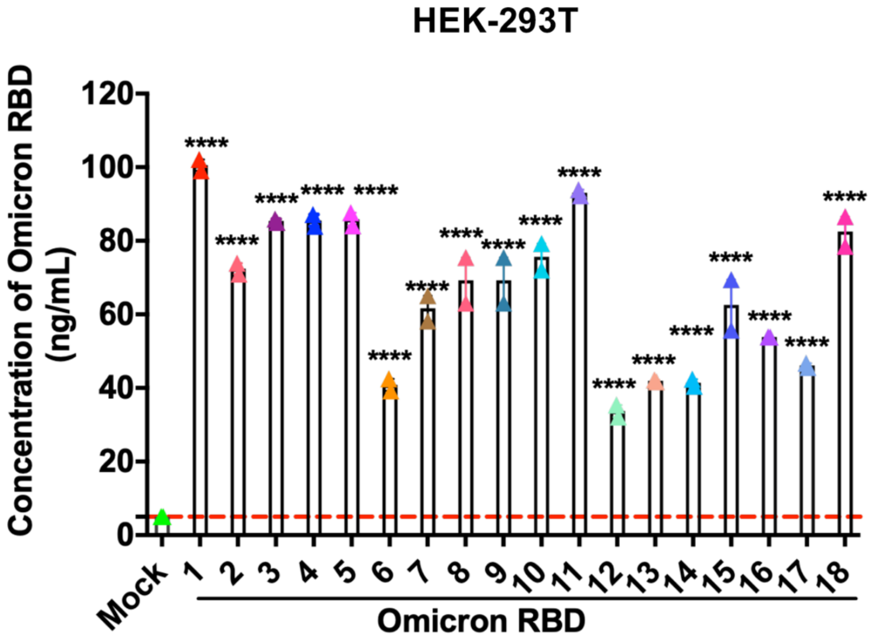

Fig. S1. Omicron RBD protein expression by mRNAs in HEK-293T cells. HEK-293T cells were transfected with 18 optimized mRNAs (5 μg/well), and the expression of Omicron RBD was measured by ELISA 15 h after transfection. The red dashed lines indicate the detection limit of the assay. Data are shown as mean ± SEM and analyzed using one-way ANOVA with multiple comparisons tests (**** $P< 0.0001$).
