# Supplementary material for: Rapid development of an updated mRNA vaccine against the SARS-CoV-2 Omicron variant
Source: Cell Res. 2022 Feb 14;32(4):401–3. doi: 10.1038/s41422-022-00626-w (PMC8853430; doi:10.1038/s41422-022-00626-w)
Supplement: Supplementary file 3 — Supplementary information, Fig. S2 [file 41422_2022_626_MOESM3_ESM.pdf]

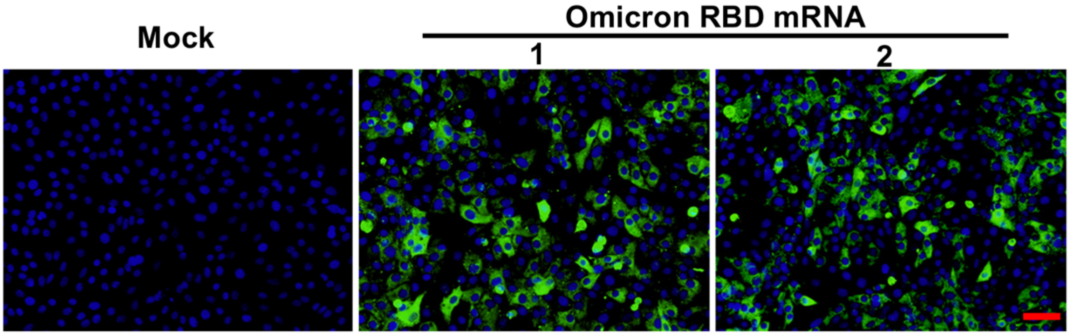

Fig. S2. Immunofluorescence staining assay for the expression of Omicron RBD. The Omicron RBD encoding mRNAs were transfected into Vero cells, and immunofluorescence staining was performed used an Omicron RBD-reactive monoclonal antibody 24 h after transfection. The Omicron RBD was stained in green, and DAPI (blue) staining indicates the nucleus. Scale bar, 100 μm.
